# Preconceptional brain structure and future conception: a prospective brain MRI study among 321 women

**DOI:** 10.1038/s41598-025-88075-4

**Published:** 2025-02-06

**Authors:** Merel W. de Vries, Eline M. P. Poels, Gennady V. Roshchupkin, Ryan L. Muetzel, Milan Zarchev, Astrid M. Kamperman, Henning Tiemeier, Meike W. Vernooij, Steven A. Kushner

**Affiliations:** 1 Department of Psychiatry, Erasmus MC, University Medical Center Rotterdam, ‘s Gravendijkwal 230, Rotterdam, 3000 CA The Netherlands; 2 The Generation R Next Study Group, Erasmus MC, University Medical Centre Rotterdam, Rotterdam, The Netherlands; 3 Department of Radiology and Nuclear Medicine, Erasmus MC, University Medical Center Rotterdam, Wytemaweg 80, Rotterdam, 3015 CN The Netherlands; 4 Department of Epidemiology, Erasmus MC, University Medical Center Rotterdam, Wytemaweg 90, Rotterdam, 3015 CN The Netherlands; 5 Department of Child and Adolescent Psychiatry, Erasmus MC University Medical Center-Sophia Children’s Hospital, Rotterdam, The Netherlands; 6 Department of Social and Behavioral Sciences, Harvard T.H. Chan School of Public Health, Boston, MA USA; 7 Department of Psychiatry, Columbia University Irving Medical Center, New York, USA

**Keywords:** Brain structure, Magnetic resonance imaging, Conception, Fertility, Pregnancy, Neuroscience, Physiology

## Abstract

Brain structure may influence female fertility through its reciprocal relationship with the endocrine system, but this hypothesis is underexplored. This study investigated the association between preconceptional brain structure and the likelihood of conception in a prospective population-based neuroimaging cohort. Women intending to conceive within a year were recruited and structural brain MRI scans were collected from 321 participants between June 2019 and March 2021. During the 12-month follow-up, 185 women became pregnant, and 136 did not. Logistic regression was used to examine associations between global brain parameters and conception, adjusting for age, intracranial volume, BMI, prior STD diagnosis, ethnicity, education, household income, smoking, and alcohol use. Unadjusted analyses showed associations between conception and larger occipital lobe and nucleus accumbens volume, increased surface area across all lobes, and occipital cortical thickness, and conception. Adjusted analyses identified a positive association between nucleus accumbens volume and conception (OR = 1.50 (95% CI: 1.12, 1.99), p-value = 0.007). Sensitivity analyses linked caudate volume to conception, but no findings remained significant after correction for multiple comparisons. Further research is needed to understand the potential role of brain structure and function in conception, in relationship with general health and socioeconomic factors.

## Introduction

There is growing evidence showcasing that pregnancy is associated with long-term changes in brain structure and function^1–13^. However, it is unknown when any changes arise. Additionally, it is unknown what role preconceptional brain structure plays in these changes over pregnancy and in the likelihood of future conception. Given the strong, reciprocal relationship between the brain and the endocrine system, it is possible that preconceptional brain structure plays a role in female fertility.

Female reproduction encompasses a multitude of processes. Several of these are determined by the reproductive organs, but there is also a central role for the brain. The reproductive system relies on a complex balance of hormones that orchestrate the menstrual cycle and influence fertility. The brain controls the production of these hormones through the gonadotropin-releasing hormone (GnRH) neuronal network^14^. This network governs the onset of puberty and subsequent fertility. The GnRH network controls secretion of gonadotropins by the pituitary gland, thereby determining patterns of pulsatile secretion of circulating luteinizing hormone (LH) and follicle-stimulating hormone (FSH), as well as the mid-cycle preovulatory surge of gonadotropins in females^15–17^. In women, the ovaries are the primary target organs for LH and FSH, which stimulate ovarian cells to secrete estrogens and progestins^18^. As such, abnormalities in the GnRH neurons themselves, or neuronal and glial cells that connect with them, can lead to defective puberty and fertility^19,20^.

In turn, it is known that sex steroid hormones exert an effect on neuronal organization and plasticity^21^. There is growing evidence that the maternal brain exhibits considerable structural plasticity in association with pregnancy and parturition^1,5,13^. Pregnancy is characterized by profound surges of sex steroid hormones. Other endocrine events involving rapid fluctuations in hormone levels such as puberty are also known to render structural alterations in the human brain^22–24^.

While a woman’s fertility depends in part on both female and male reproductive physiology, these are not the only determinants. Reproductive behavior is essential and highly linked to sociocognitive development. For example, in adolescence, hormonal development and variations during puberty are associated with intensified social behaviors such as aggression and risk-taking^25^. These hormonal changes are also linked to increased socio-affective reactivity, including heightened sensitivity to social rejection and the motivational importance of social status^26^. Furthermore, they impact sociocognitive abilities, such as the capacity to infer complex social emotions^27^. Physiological changes associated with puberty also contribute to enhanced functional connectivity within the mentalizing network of the social brain^28^. Consequently, these physiological changes and associated behavior may influence a woman’s likelihood of conception through effects on reproductive behavior.

There is thus a strong, reciprocal relationship between the brain and the endocrine system. Based on the governing role of the brain in the production of sex steroids, and in turn the influence of sex steroids on the neuronal organization of the brain, preconceptional brain structure might be associated with a woman’s future likelihood of conception. Yet, to our knowledge, no prior studies have investigated the association between human brain structure and likelihood of conception.

We performed a prospective longitudinal population-based brain MRI study among 321 preconceptional women who were followed-up for occurrence of pregnancy over a 12-month period.

## Methods

### Study design and participants

Participants were enrolled in a population-based prospective cohort, the Generation R *Next* Study, from fetal life until adulthood in Rotterdam, the Netherlands. The cohort is designed to enable the identification of early environmental and genetic causes and causal pathways leading to normal and abnormal growth, development and health. Women who were pregnant or planning on getting pregnant within the next year between August 2017 and March 2021 were eligible for participation in Generation R *Next*. Participants were recruited through letters and public advertisements. For the current study, brain MRI scans were acquired in a subsample of women, between June 2019 and March 2021. All pre-enrolled and newly registered preconceptional participants were asked to undergo a brain MRI scan. Women were excluded if they were pregnant at the time of the study visit, continued to use contraceptives across the follow-up period, and/or had any contra-indications for undergoing magnetic resonance imaging. Participants were scanned preconceptionally and followed-up for occurrence of pregnancy for a minimum of 12 months.

The study was approved by the Medical Ethics Committee of Erasmus Medical Center (MEC2016-589). All research was conducted in accordance with the relevant ethical guidelines and regulations, including the Declaration of Helsinki. Written informed consent was obtained from all participants before their participation in the study.

### Definition of conception

Conception was defined as a positive pregnancy test (regardless of pregnancy outcome) within 12 months following the preconceptional brain imaging study visit. Participants were asked to contact the research center when they had a positive pregnancy test, preferably before the seventh week of gestation. All participants were recontacted for follow-up one year after the baseline study visit.

### Magnetic resonance imaging

MRI data were acquired on a 3 Tesla GE Discovery MR750w system using an eight-channel receive-only head coil (General Electric, Milwaukee, WI). High resolution T1-weighted images were acquired using a 3D coronal inversion recovery fast spoiled gradient recalled (IR-FSPGR, BRAVO) sequence with the following parameters: repetition time (TR) = 8.77 ms, echo time (TE) = 3.4 ms, inversion time = 600 ms, flip angle = 10°, field of view = 220 × 220 mm, matrix = 220 × 220, ARC imaging acceleration factor of 2, slice thickness = 1.0 mm, number of slices = 230, and an in-plane resolution of 1.0 mm^2^.

### Image quality control and processing

The FreeSurfer image analysis suite version 6.0 was used to perform automated cortical reconstruction and volumetric segmentation (http://surfer.nmr.mgh.harvard.edu/)^29^. The steps included: removal of non-brain tissue (e.g., skull strip), voxel intensity normalization, initial tissue segmentation, cortical reconstruction and automated anatomical labeling. Anatomical labeling was conducted using the Desikan-Killiany Atlas^30^.

Quality of the T1-weighted images was visually inspected immediately upon acquisition in a systematic manner by a trained rater who was blind for the outcome. If the quality was not deemed acceptable, the T1-weighted scan was rerun during the same MRI visit. After FreeSurfer processing, further quality control was done in line with previously described procedures^31^. In cases where cortical reconstructions were inappropriate (*N* = 74), manual corrections using gray matter control points were added to improve white and gray matter surface reconstructions. In all cases, the control points significantly improved reconstructions. Consequently, no scans were excluded from analysis. All scans were examined by a clinical neuroradiologist for the presence of incidental findings of potential clinical relevance. One clinically relevant incidental finding was reported in the brain imaging data. This participant did not need to be excluded from the analysis, since the finding did not impact automated cortical reconstruction and volumetric segmentation.

### Brain morphology parameters

We were interested in the association between preconceptional brain structure and conception after 12 months of follow-up. To reduce the risk of multiple testing, we selected global brain parameters (total gray matter, total white matter, lateral ventricles and volume, surface area and cortical thickness for the frontal, parietal, temporal and occipital lobes) and subcortical volumes (thalamus, amygdala, hippocampus, putamen, pallidum, caudate, accumbens) for our regions of interest analysis.

### Statistical analyses

#### Logistic regression analyses

All analyses were performed using the Statistical Package for the Social Sciences (SPSS, version 26, IBM). Demographic characteristics are reported in accordance with the STROBE guidelines^32^.

Separate logistic regression models were performed using structural brain parameters as independent variables and conception (dichotomous) within a 12-month follow-up as dependent variable. Structural brain parameters were standardized into z-scores, to enhance comparison between the odds ratios. Based on the literature on fertility we selected several key factors as covariates for our models. Factors were considered potential confounding factors if they were strongly associated with female fertility^33,34^ and were associated with brain structure^35–38^. Adjustment for potential confounding variables was performed using a two-step procedure. In Model I, age and intracranial volume (ICV) were included as covariates. Model II expanded on Model I by adding Body Mass Index (BMI), prior diagnosis of a sexually transmitted disease (STD), ethnicity, education level, household income, smoking, and alcohol consumption as additional covariates. In sensitivity analyses, cases with a diagnosis of primary ovarian insufficiency (POI) or polycystic ovarian syndrome (PCOS) were excluded from the fully adjusted analysis to assess whether results were driven by diagnosed fertility disorders. Given recent evidence of volumetric brain changes across the menstrual cycle^39,40^, we conducted a sensitivity analysis to assess whether the menstrual cycle phase at the time of the MRI scan influenced our results. To account for this potential effect, the number of days since the start of the last menstruation was included as a covariate in Model II.

To control for Type-I errors, we applied a false discovery rate (FDR) correction on the total of 22 brain parameters that were tested. FDR corrected p-values < 0.05 were considered statistically significant. In text and tables nominal p-values are presented and marked if they were statistically significant after FDR correction. Missing data on covariates were handled using multiple imputation with 20 iterations.

## Results

### Sample characteristics

**Fig. 1:**
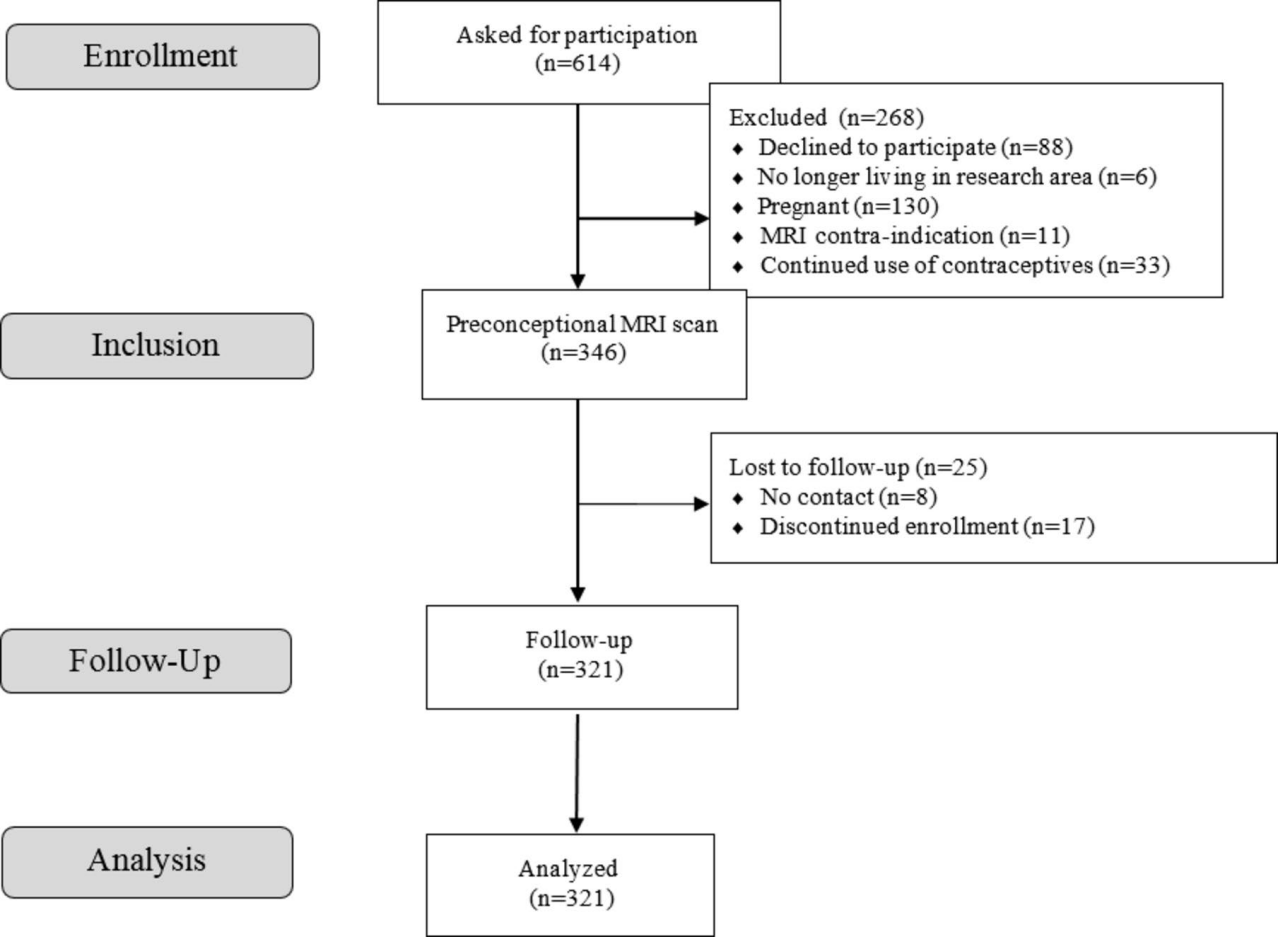
Flow chart of the study population.

In total, 614 participants enrolled in the Generation R *Next* Study between June 2019 and March 2021 were asked to participate in the brain imaging study. Of these, 88 participants declined to participate and 130 participants were already pregnant. A total of 346 participants were included in the study and underwent brain MRI. Twenty-five participants were lost to follow-up with no data on pregnancy outcome. A total of 321 participants were included in the final analysis (Fig. 1). Of these, 185 women became pregnant within the 12 months follow-up and 136 did not become pregnant. The mean age of the conception cohort at inclusion was 31.7 years (SD 3.4, range 20.7–41.8) and that of the no conception cohort was 33.2 years (SD 5.3, range 20.1–48.8). On average, women who became pregnant were younger, higher educated, and more likely of Dutch descent and nulliparous. Demographic and obstetric characteristics are reported in Table 1.

**Table 1:** Demographic and clinical characteristics of the study sample at inclusion into the study^a^.

	Conception	No conception
N	185	136
Age at MRI in years, mean (SD)	31.7 (3.4)	33.2 (5.3)
Nulligravid, n (%)	129 (72.5)	74 (66.1)
Nulliparous, n (%)	150 (83.3)	95 (81.9)
Dutch ethnicity, n (%)	127 (69.8)	54 (41.5)
Net household income per month > 4000 euro, n (%)	125 (69.8)	55 (43.3)
Higher level of education, n (%)	144 (80.4)	94 (72.9)
BMI in kg/m^2^, mean (SD)	24.0 (4.0)	25.7 (5.9)
Currently smoking cigarettes, n (%)	9 (5.3)	9 (8.0)
Alcohol consumption of one or more units per week, n (%)	101 (60.8)	50 (44.6)
Diagnosis of POI, n (%)	0 (0.0)	1 (0.9)
Diagnosis of PCOS, n (%)	11 (6.1)	7 (6.2)
Prior diagnosis of STD^b^, n (%)	37 (20.6)	39 (34.5)
Days since last menstruation, mean (SD)	14.1 (29.1)	13.2 (14.8)

^a^ In case of missingness valid means and percentages are reported. There were missing observations for ethnicity (*N* = 9 (2.8%), conception: *N* = 3, no conception *N* = 6), household income (*N* = 15 (4.7%), conception: *N* = 6, no conception: *N* = 9), education level (*N* = 13 (4.0%), conception: *N* = 6, no conception: *N* = 7), gravidity (*N* = 31 (9.7%), conception: *N* = 7, no conception: *N* = 24), parity (*N* = 25 (7.8%), conception: *N* = 5, no conception: *N* = 20), BMI (*N* = 5 (1.6%), conception: *N* = 2, no conception: *N* = 3), smoking (*N* = 39 (12.1%), conception: *N* = 15, no conception: *N* = 24), alcohol consumption (*N* = 43 (13.4%), conception: *N* = 19, no conception: *N* = 24), diagnosis of POI or PCOS (*N* = 28 (8.7%), conception: *N* = 5, no conception *N* = 23), diagnosis of STD (*N* = 28 (8.7%), conception: *N* = 5, no conception: *N* = 23), days since last menstruation (*N* = 14 (4.3%), conception *N* = 10, no conception *N* = 4).

^b^ Specific diagnoses of STD per group. Conception: Chlamydia (*N* = 27), Genital Warts (*N* = 9), Gonorrhoea (*N* = 3), Herpes Genitalis (*N* = 5), Other STD (*N* = 1), No Conception: Chlamydia (*N* = 29), Genital Warts (*N* = 7), Gonorrhoea (*N* = 3), Herpes Genitalis (*N* = 5), Other STD (*N* = 2). Some women reported multiple diagnoses.

BMI: Body Mass Index, POI: primary ovarian insufficiency, PCOS: polycystic ovarian syndrome, STD: sexually transmitted disease.

### Individual global and subregional logistic models

Table 2 provides the results of the minimally adjusted logistic regression analyses. Model I showed that larger occipital lobe volume (OR per z-score = 1.36 (95% CI: 1.02, 1.82), p-value = 0.04) and nucleus accumbens volume (OR = 1.31 (95% CI: 1.01, 1.70), p-value = 0.04) were associated with a higher likelihood of conception.

Additionally, larger surface area of frontal lobe (OR = 1.48 (95% CI: 1.16, 1.89), p-value = 0.002), parietal lobe (OR = 1.31 (95% CI: 1.03, 1.67), p-value = 0.03), temporal lobe (OR = 1.49 (95% CI: 1.16, 1.91), p-value = 0.002) and occipital lobe (OR = 1.38 (95% CI: 1.09, 1.75), p-value = 0.008) related to a higher chance of conception.

Lastly, larger cortical thickness in the occipital lobe (OR = 1.33 (95% CI: 1.06, 1.68), p-value = 0.02) positively related to conception. Importantly, associations with frontal lobe surface area and temporal lobe surface area remained statistically significant after FDR correction.

After correction for potential confounders (Model II), only the association with accumbens volume (OR = 1.50 (95% CI: 1.12, 1.99), p-value = 0.007) persisted. This association was not statistically significant after FDR correction.

After excluding cases with a diagnosis of PCOS or POI there was also an association between a larger caudate volume and a higher chance of conception within 12 months follow-up (OR = 1.53 (95% CI: 1.09, 2.14), p-value = 0.02) after correction for potential confounders. This was not statistically significant after FDR correction. Other results did not change in sensitivity analyses. Adding the number of days since the start of the last menstrual period as a variable to Model II did not change the results.

**Table 2:** Associations between z-scores of brain parameters and conception during 12 months follow-up.

Brain volume parameters	Model I^a^		Model II^b^	
	OR (95% CI)	*p*-value	OR (95% CI)	*p*-value
Total gray matter	1.59 (0.96, 2.61)	0.07	1.35 (0.80, 2.30)	0.26
Total white matter	0.90 (0.62, 1.30)	0.56	0.99 (0.66,1.48)	0.96
Lateral ventricle	0.88 (0. 68, 1.14)	0.32	0.88 (0.67, 1.16)	0.37
Frontal lobe	1.11 (0.75, 1.64)	0.62	1.07 (0.70, 1.63)	0.76
Parietal lobe	1.03 (0.70, 1.51)	0.89	1.00 (0.67, 1.50)	0.98
Temporal lobe	1.33 (0.90, 1.98)	0.15	1.28 (0.84, 1.94)	0.26
Occipital lobe	1.36 (1.02, 1.82)	0.04	1.24 (0.91, 1.69)	0.18
Thalamus	1.00 (0.72, 1.39)	0.99	0.99 (0.71, 1.40)	0.97
Amygdala	1.03 (0.79, 1.35)	0.82	1.09 (0.82, 1.45)	0.55
Hippocampus	1.25 (0.93, 1.66)	0.14	1.20 (0.88, 1.63)	0.25
Putamen	0.91 (0.70, 1.18)	0.49	1.01 (0.76, 1.33)	0.96
Pallidum	0.85 (0.65, 1.11)	0.23	0.91 (0.68, 1.21)	0.51
Caudate	1.31 (0.99, 1.75)	0.06	1.35 (0.99, 1.83)	0.06
Accumbens	1.31 (1.01, 1.70)	0.04	1.50 (1.12, 1.99)	0.007
Surface area				
Frontal lobe	**1.48 (1.16**,** 1.89)**	0.002*	1.24 (0.95, 1.62)	0.12
Parietal lobe	1.31 (1.03, 1.67)	0.03	1.15 (0.88, 1.50)	0.31
Temporal lobe	**1.49 (1.16**,** 1.91)**	0.002*	1.30 (0.99, 1.71)	0.06
Occipital lobe	1.38 (1.09, 1.75)	0.008	1.26 (0.97, 1.64)	0.08
Cortical thickness				
Frontal lobe	0.88 (0.67, 1.11)	0.28	0.94 (0.74, 1.21)	0.63
Parietal lobe	1.22 (0.96, 1.53)	0.10	1.12 (0.87, 1.44)	0.38
Temporal lobe	1.10 (0.87, 1.38)	0.42	1.08 (0.84, 1.38)	0.55
Occipital lobe	1.33 (1.06, 1.68)	0.02	1.12 (0.87, 1.45)	0.37

OR = odds ratio, CI = confidence interval.

^a^Model I: adjusted for age and total intracranial volume (surface area and cortical thickness were not adjusted for intracranial volume).

^b^Model II: adjusted for age, total intracranial volume, prior diagnosis of STD, BMI, ethnicity, education level, household income, smoking, and alcohol consumption.

*Statistically significant after FDR correction.

## Discussion

Our results indicate that global brain metrics do not have strong associations with future conception across the 12 months follow-up period. Only the nucleus accumbens volume showed an association with the likelihood of conception; women who became pregnant had a larger volume, although not statistically significant after FDR correction. Our minimally adjusted findings suggest that brain structure may be an indicator of health, socioeconomic circumstances, social behavior, or lifestyle which collectively determine the likelihood of conception.

The positive association of nucleus accumbens volume with likelihood of conception requires further investigation, as this may reflect a particular neural pathway related to fertility. The nucleus accumbens is a key component of the brain’s reward system. Dopamine release in the nucleus accumbens is important for the assignment of motivational value to rewarding behaviors^41^, which are essential to reproduction^42^. In addition, research in both animals and humans has demonstrated a connection between pregnancy and the nucleus accumbens^40–43^. In rodent mothers, cues from offspring carry significant reinforcing value, motivating maternal care. This effect is mediated by dopamine release into the nucleus accumbens^43–45^. A similar association has been identified in human mothers^46^. Based on these findings, one could speculate that the nucleus accumbens also plays a role in the likelihood of conception by influencing reproductive behavior, motivation, and decision-making^47^.

A major strength of our study is its large sample size and prospective design. Because of the recruitment strategy of the population-based cohort study that this project was embedded in, we were able to recruit a large sample of women from the Dutch population. Women were followed for the occurrence of pregnancy over a minimum of 12 months. Additionally, because of the high quality of the imaging data collected, no scans had to be excluded. Lastly, the design of the study is unique. This is, to our knowledge, the first study conducted assessing preconceptional brain structures in a cohort of women trying to conceive.

The results of this study also need to be interpreted in the context of several limitations. We do not have information on how long women had already been trying to conceive prior to their enrollment in the study. All participants were followed up for a minimum of 12 months, but it is possible that some had been trying conceive long before they were enrolled. Moreover, no information on male fertility was available in this study. Additionally, only T1-weighted scans were analyzed for the purpose of this study. It is possible that other MR sequences could provide more insight into the physiology of the observed differences. In particular, functional brain imaging could shed light on the behavioral component. Future research should try to incorporate other factors known to influence fertility, and possibly physiological markers of female and male fertility, such as reproductive hormone levels, to gain a more in-depth understanding of the association between preconceptional brain structure and conception.

In conclusion, the current findings do not indicate a robust relationship between preconceptional global brain parameters and the chance of conception within a 12-month follow-up. The nucleus accumbens finding requires further investigation. These data provide, to our knowledge, the first insights into the association between preconceptional human brain structure and a woman’s likelihood of future conception.

## Data Availability

Data from this study are available upon reasonable request to the director of the Generation R Study (generationr@erasmusmc.nl), subject to local, national and European rules and regulations.

## Declarations

### Attestation statements

Data regarding any of the participants in the study have not been previously published unless specified. Data will be made available to the editors of the journal or review or query upon request.

### Capsule

No direct association between global brain parameters at preconception and future conception during 12-month follow-up was observed. Further investigation of the role of the nucleus accumbens in female fertility is warranted.
